# Assessing Barriers to Human Papillomavirus (HPV) Vaccination in At-Risk Rural Communities of Western North Carolina, United States

**DOI:** 10.3390/vaccines11121785

**Published:** 2023-11-29

**Authors:** Candice J. McNeil, Breona Barr, Iqra Munawar, Michael E. DeWitt, Jenny Snow Myers, Avinash K. Shetty

**Affiliations:** 1 Office of Global Health, Wake Forest University School of Medicine, Medical Center Boulevard, Winston-Salem, NC 27157, USA; breona.barr@hsc.wvu.edu (B.B.); ashetty@wakehealth.edu (A.K.S.); 2 Section on Infectious Diseases, Wake Forest University School of Medicine, Winston-Salem, NC 27157, USA; imunawar218@gmail.com (I.M.); medewitt@wakehealth.edu (M.E.D.); 3 Department of Family Medicine, West Virginia University, Ranson, WV 26506, USA; 4 Department of Biology, Wake Forest University, Winston-Salem, NC 27109, USA; 5 Immunization Branch, Division of Public Health, Carolina Department of Health and Human Services, Raleigh, NC 27609, USA; jenny.myers@dhhs.nc.gov

**Keywords:** HPV, vaccine, rural, barriers, adolescents

## Abstract

Human papillomavirus (HPV) vaccination among adolescents in rural, western North Carolina (NC) remains suboptimal. Data are needed to understand the barriers to HPV vaccination in these communities. We conducted a cross-sectional pilot study of parental attitudes and provider practices regarding HPV vaccination in rural western NC counties with lower HPV vaccination rates. Eight health department clinics were enrolled in the study. Further, 29 provider and 32 parent surveys were analyzed along with environmental scans. Median provider comfort regarding knowledge of HPV-associated diseases was 85% (IQR = 75–95), on a scale of 0–100% (100% representing complete comfort). Median parental comfort level regarding knowledge of HPV-associated diseases and the HPV vaccine was 75% (IQR = 50–89) and 75% (IQR = 49–96), respectively. Less than 25% of parents rated the HPV vaccine as ‘extremely effective’ against genital (16.7%) and anal cancers (17.9%). Parents were more likely to rate the vaccine as ‘extremely effective’ to ‘very effective’ if their child was female. There was no significant difference between parental- and provider-reported comfort with knowledge about HPV-associated diseases (*p* = 0.0725) and the HPV vaccine (*p* = 0.167). This study identified multiple opportunities to increase HPV vaccine coverage among unvaccinated adolescents at parental, provider, and clinic levels. Health education of rural NC residents and providers in public health settings may identify future interventions to increase HPV vaccine uptake.

## 1. Introduction

Human papillomavirus (HPV) remains the most common sexually transmitted infection (STI) in the United States (US) [1]. A vaccine against four and, later, nine HPV strains most often associated with genital warts and genital cancers has been approved since 2006 and 2014, respectively [2]. The three-dose series was modified in 2016 to allow two-dose scheduling in children 9–14 years of age, when the vaccine is most immunogenic [3]. Because exposure to HPV occurs early, often at the time of sexual debut [4], early vaccination is key for both the prevention and increased likelihood of series completion. Longitudinal data have shown decreased prevalence of HPV types associated with the vaccine at the population level; however, variable uptake in rural communities, for example, has influenced local outcomes [5].

**Figure 1:**
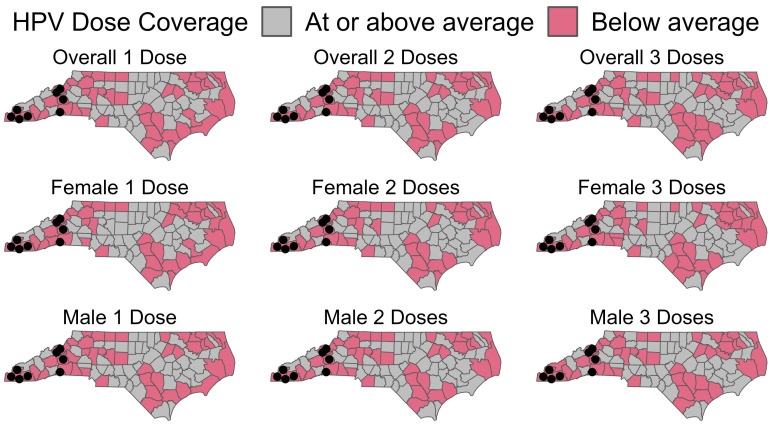
Overview of counties in North Carolina below the state average rate for HPV vaccines in 2017, by dose coverage and sex. Black dots represent the proposed survey locations. Data provided by the NC Immunization Branch.

Uptake of the HPV vaccine continues to fall behind national goals to increase series completion in male and female adolescents [6]. In 2019, national coverage for ≥1 dose of the HPV vaccine among adolescents 13–17 years old in the U.S was 71.5%, with series completion at 54.2 [7]. Publicly funded vaccine services are provided for North Carolina’s 100 counties via 86 local health departments or districts that support multiple counties. In June of 2017, none of the North Carolina counties were meeting Healthy People 2020 goals for HPV vaccination. In North Carolina (NC), overall first-dose vaccination rates were comparable at 71.3%; however, series completion was lower than the national coverage at 49.5% [7]. In 2017, overall vaccination coverage rates for all North Carolina residents aged 13–17 were 45.6, 33.9, and 24.5 for dose 1, dose 2, and dose 3, respectively. However, there is some heterogeneity with rates of 49.3, 37.9, and 27.7 for dose 1, dose 2, and dose 3, respectively, among females and 42.8, 30.8, and 21.1 for dose 1, dose 2, and dose 3 among males, respectively. Western NC is a mountainous region and is home to the Appalachian Mountains. This area is characterized by few urban or commercial centers and relatively low population densities, leading to a high spatial variability in coverage (Figure 1). Therefore, we selected 10 North Carolina counties as candidates for deploying the survey instrument. The overall vaccine coverage in these counties ranges from 24.4 to 43%, 15.3 to 31.4, and 10.1 to 21.7% for dose 1, dose 2, and dose 3, respectively. 

The barriers affecting rates of HPV vaccine initiation and completion can exist at the parent, provider, and clinic levels. Parental knowledge, attitudes, and beliefs (KAB) about the vaccine and sociodemographic variables may influence rates of acceptance and completion in minors [8]. Healthcare providers in the community are uniquely positioned to positively influence HPV vaccine coverage. Data from Walker et al. [9] highlighted the importance of provider recommendation in promoting vaccination coverage. In this report, HPV vaccine coverage among adolescents without reported provider recommendation was 46.7%, versus 74.7% among those with reported provider recommendation [9]. In NC, coverage increased from 50.5% to 76.2% in adolescents reporting provider recommendation [9]. System-level barriers that may complicate immunization efforts include consent requirements or the presence of gender or age bias in recommendations, leading to missed vaccination opportunities [8,10].

Parental KAB, social factors, and lack of provider recommendation may also represent a combined effect on vaccine uptake [11]. Data from the 2017–18 National Immunization Survey (NIS-Teen) showed HPV vaccine hesitancy, primarily due to safety concerns, as a major contributing factor in lack of intent to initiate the HPV vaccine in adolescents, while lack of provider recommendation contributed significantly to a lack of series completion [12]. Our pilot study aimed to gather data on parental KAB and healthcare practices specific to rural communities in NC reliant on public health departments for vaccine services to aid in the development of effective methods to increase local rates of HPV vaccine uptake and series completion.

## 2. Materials and Methods

We conducted a cross-sectional pilot study of parental KAB about the HPV vaccine and healthcare provider practices in rural Western NC Local Health Department (LHD) clinics. Ten Western NC counties with HPV vaccination rates lower than the state average were invited to participate in the study. Vaccine Coordinators and/or Supervisors in each clinic were contacted for study approval and coordination.

Clinic and provider practices were assessed using surveys adapted from the AFIX (Assessment, Feedback, Incentives, and exchange) questionnaire [13]. Providers were defined as those with patient contact sufficient to influence parental KAB, including front-desk positions, medical assistants, nurses, physicians, etc. As the infrastructure of the vaccine programs was unknown, the Vaccine Coordinator and/or Supervisor served as the initial contact to share the electronic survey link with clinic staff involved in vaccine services. This snowball sampling method was utilized given the known effectiveness in identifying populations that are difficult to access [14]. Environmental scans were conducted to observe patient flow, immunization practices, and interview providers at each site.

Parental views were assessed with modified versions of the Carolina HPV Immunization Attitudes and Beliefs Scale (CHIAS) [15,16]. Parents and legal guardians (collectively referred to as ‘Parents’) of minors utilizing clinic services were identified by simple random selection and invited to complete surveys on an electronic tablet provided by the study team. A simple random number generator was provided to each site for randomization procedures. Clinics were also granted flexibility in incorporating survey distribution into their workflow.

Institutional Review Board approval was obtained for this study. As a primarily descriptive pilot study, no formal sample size calculations were performed. However, stopping rules for sample collection and scoping were generated. Collection of 5 parent and 5 provider surveys, for a total of 10 surveys per site, was initially approved. Subsequent approval to allow collection of up to 20 surveys was later obtained to accommodate sites able to collect additional surveys. Collection was considered complete, however, once 5 of each survey type were received or date collection concluded, whichever came first. The Mann–Whitney test with an alpha level of 0.05 was used to determine if there was a significant difference between the comfort level of parents and the provider regarding knowledge of HPV-associated disease and the HPV vaccine. Data were managed utilizing REDCap (Research Electronic Data Capture) tools [17]. This research was approved by the institutional review board of Wake Forest University School of Medicine.

## 3. Results

Survey collection and site visits were conducted from September 2019 to April 2020 in eight counties: Clay, Cherokee, Graham, Macon, McDowell, Mitchell, Polk, and Yancy. Over the span of the study, a total of seven environmental scans were performed. Surveys were collected via electronic link and tablet, with paper forms as a backup method. Further, 32 parent and 29 provider surveys were received. One county participated in a site visit and submitted provider surveys prior to data capping. Graham and McDowell county sites did not randomize parent enrollment due to low patient volume. Due to coronavirus restrictions, other administrative barriers, or study closure, Yancy county clinic completed one provider survey without parental survey or environmental scan, Mitchell county clinic completed one environmental scan without provider or parent surveys, and Polk county clinic completed the environmental assessment by phone.

**Figure 2:**
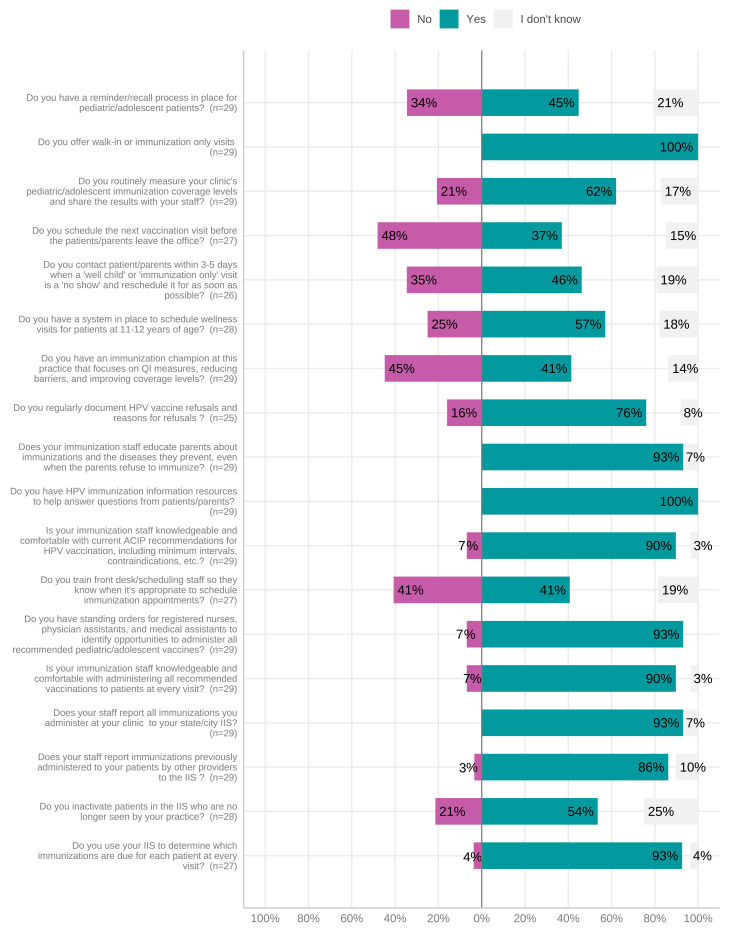
Provider-reported clinic practices for immunization appointments. Abbreviations: HPV, human papillomavirus.

Our study fairly well represents the race and ethnicity for the surveyed counties’ demographics of 5.1% Hispanic or Latino and 93.2% white; however, our survey respondents skewed more female than the 51% female population estimate based on 2017 American Community Survey estimates. The characteristics of providers and parents are shown in Table 1. The reported characteristics of Western NC clinics are shown in Table 2. All clinics participated in the Vaccines for Children (VFC) Program, which provides vaccines at no cost to eligible children, and utilized the NC Immunization Registry (NCIR) to report and screen for missing immunizations. Two counties utilized satellite clinics in or near local high schools for use by students with parental permission. Figure 2 displays the clinic immunization practices reported by providers. Median provider comfort regarding knowledge of HPV-associated diseases was 85% (IQR = 75–95), on a scale of 0 to 100% (with 100% representing complete comfort). Median provider comfort with knowledge about the HPV vaccine and vaccine resources was 85% (IQR = 75–95) and 85% (50–95), respectively.

**Figure 3:**
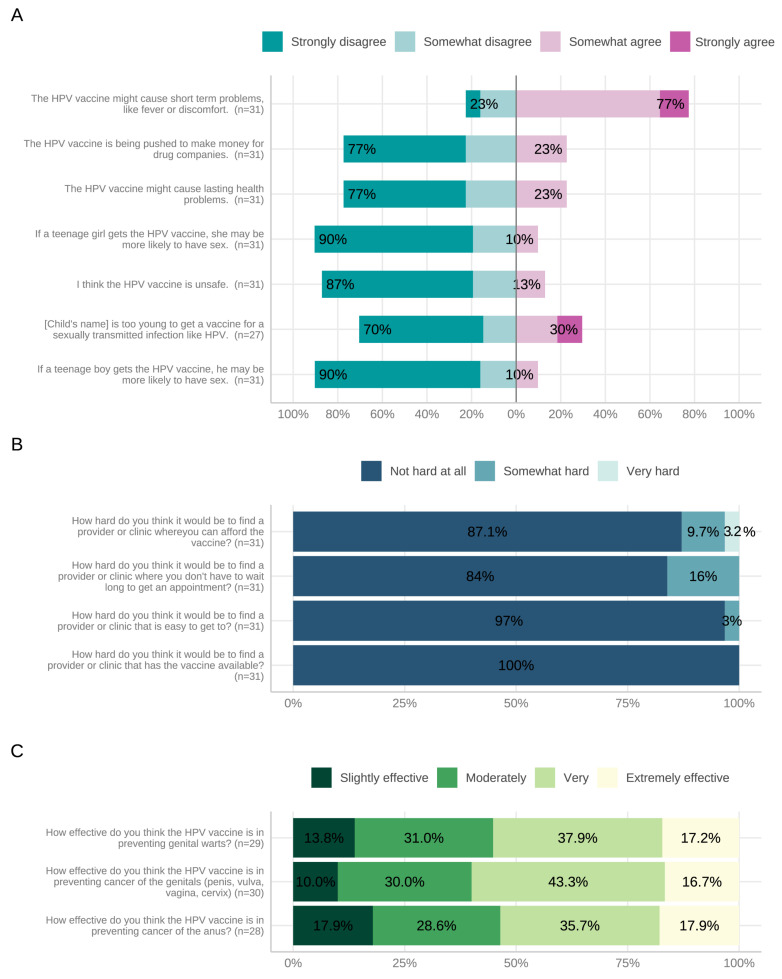
Parent-reported knowledge (**A**), barriers (**B**), and attitudes (**C**) to HPV vaccination. Abbreviations: HPV, human papillomavirus.

Figure 3 summarizes parental KABs and barriers to HPV vaccination. Less than 25% of parents rated the HPV vaccine as ‘extremely effective’ against genital (16.7%) and anal cancers (17.9%) (Figure 3). Parents were generally more likely to rate the vaccine as ‘extremely effective’ to ‘very effective’ if their child was female (Table 3). The median parental comfort level regarding knowledge of HPV-associated diseases and the HPV vaccine was 75% (IQR = 50–89) and 75% (IQR = 49–96), respectively. There was no significant difference between parental- and provider-reported comfort with knowledge about HPV-associated diseases (*p* = 0.0725) and the HPV vaccine (*p* = 0.167).

## 4. Discussion

Our study, while designed as a descriptive pilot study, identified multiple opportunities to increase HPV vaccine coverage at parental, provider, and clinic levels within the counties surveyed, which can be used to design interventions and educational products to increase HPV vaccine uptake. Gaps in knowledge about the HPV vaccine were a major theme among those surveyed. Many parents felt their child was too young for the HPV vaccine or wanted more information before deciding, suggesting that education could have a significant impact on HPV vaccination rates and potentially reduce the risk of delayed vaccination. In the NIS-Teen survey, knowledge gaps and the belief that vaccines are not needed or unnecessary were common reasons for lack of parental intent to initiate and complete the HPV vaccine series [12]. Our study also identified that given the limited number of visits these patients had to these clinics, with a median of two, it is essential that the providers are equipped to both provide the vaccine and educate patients. Furthermore, this study highlighted that half of responding providers did not schedule future vaccines at the current visit, nor have immunization champions identified with associated quality improvement methods, representing potential missed opportunities and operational blind spots.

Initiation of the HPV vaccine series after age 15 forfeits the two-dose schedule, potentially creating an additional barrier to series completion [18]. In North Carolina, supported by NC G.S. 90–21.5, minors can consent to preventative services for STIs [19], which may extend to the HPV vaccine; however, it is unclear to what extent this awareness exists. Parents are often integrally involved in facilitating visits to providers; therefore, a lack of parental support of HPV vaccination may be a system-wide barrier to adolescent immunization. Caregiver influence and the social support for caregivers can impact vaccination practices in rural populations [20]. Key stakeholders have identified multimodal approaches to HPV vaccine information presentation and dissemination, such as collaborating with school health support and further parental education to promote vaccine efforts in rural communities [21]. A recent cluster randomized trial by Dixon et al. [22], involving an educational intervention using a digital video on HPV vaccination targeting parents of HPV vaccine-eligible adolescents, found that the number of adolescents with a change in vaccination status was higher in the intervention clinic. Adolescents had greater odds of receiving a dose of the HPV vaccination when their parents had watched the video [22]. These findings underscore the role of education in vaccine behavior changes.

Nearly half of surveyed parents agreed that HPV vaccination was a safe and effective means of protecting their child from HPV-associated diseases. Similarly, 60% reported neighbors getting the HPV vaccine for their children. These data are promising, as studies suggest geospatial clustering of vaccination rates is associated with increased rates in adjacent areas, particularly for males [23]. Designing interventions to increase HPV vaccination rates in rural counties could positively influence HPV vaccine uptake in adjacent communities. In the NIS-Teen survey, parents of unvaccinated adolescents cited safety concerns as the most common reason (23%) for lack of intent to initiate the HPV vaccine series by parents [12]. More data are needed to better understand the specific reasons for lower rates in these communities. The study sites had an identifiable vaccine champion. The use of a provider champion is an effective method to implement HPV vaccination efforts in adolescents in publicly funded clinics [24]. In close-knit rural communities, relationships with providers may play a crucial role in health promotion. Many staff reported that their small clinic size allowed them to develop relationships with patients and their families and provide counseling over multiple visits. As providers may independently influence HPV vaccine uptake, enhanced provider education to address specific concerns may be beneficial [25]. High-quality targeted recommendations by providers related to cancer prevention (with emphasis on the urgency) may improve HPV vaccine uptake [26]. Similar to Zhu et al. [27], we note that continued efforts are needed to address attitudinal barriers to HPV vaccination. Enhanced provider education may be an important tool to promote effective HPV vaccination communication in well-child settings [28]. Surveyed parents were more likely to rate the vaccine as ‘extremely effective’ to ‘very effective’ if their child was female. The overall trend in parental beliefs regarding the effectiveness of the vaccine did not differ by the sex of the child in terms of significance. This is an area that warrants further exploration as there are data indicating that HPV vaccine series initiation occurs less often in male versus female children [29]. Knowledge gaps related to the risk of acquiring HPV and understanding the impact of HPV-associated cancers may influence parental gender-specific HPV vaccine beliefs and practices [29,30]. Well-designed gender-neutral messaging may promote HPV vaccine equity [31]. Additionally, obtaining data about competing vaccination messaging and motivations behind parental refusal, for example, may help identify areas to promote and support provider recommendations.

Streamlined approaches to appointment scheduling and visit reminders, as reported in provider surveys, represent opportunities to increase vaccine-positive interactions in the clinic and limit missed vaccination opportunities. The availability of walk-in immunization visits likely affected the consistency of responses to questions about follow-up appointments, contacting patients after missed appointments, and reminders. Walk-in immunizations increase convenience but may circumvent appointment reminder or tracking systems. The use of alternate modalities, such as text messaging and email, to issue reminders about upcoming immunizations that include the HPV vaccine may be beneficial, especially among age groups less likely to visit the clinic at regular intervals. The introduction of HPV vaccine mandates has been effective to overcome parental hesitancy to vaccination and improve coverage in certain states (e.g., District of Columbia, Rhode Island) [12].

This pilot study has several limitations. Convenience samples were used for recruitment in this descriptive study; as such, the participants, though similar in some aspects of demography such as race, may not be representative of the larger population. Data on the preferred language of survey participants and reasons for survey declination were not collected. As surveys were available exclusively in English, our study population may not have included participants with limited English literacy. Persons who participate in surveys may represent a unique subset of clinic attendees who may have stronger opinions more generally. These clinics serve populations that might be more inclined to vaccination as well as those more hesitant. Though surveys were intended to be administered randomly, low-volume health departments did not always meet this requirement. Less male compared to female parents participated in this survey, and it is possible that this may have influenced our results. Potential gender bias associated with HPV vaccine uptake has been well described with low-risk recognition for males and safety concerns for females and males in the setting of anti-vaccine misinformation via social media [32,33]. Gender-neutral state mandates and messages from healthcare providers and pharmaceutical industries are important strategies to improve the vaccination uptake rate [32]. Recent U.S. survey data from the 2012–2018 National Immunization Survey (NIS)–Teen show that lack of intent to initiate the HPV vaccine series increased among parents of male adolescents (from 44% in 2012 to 59% in 2018) and female adolescents from 54% to 68%, respectively. These findings indicated the need to assess the effectiveness and quality of HPV vaccine recommendations by healthcare providers [34]. Responses may be socially desirable and prone to recall bias. Selection bias is also possible in our health department-based clinic settings. Many barriers to the access of HPV vaccines have been reported in the rural southeast region of the U.S., with lower odds for HPV vaccine uptake for adolescents living at or above the poverty line, home/online (vs. public) schooling, and caregivers’ working status [35]. Potential opportunities to increase HPV vaccine uptake may involve a school-based location for HPV vaccination delivery for adolescents [36]. Future directions may include studies of other non-clinic or less traditional vaccination venues, which may increase access to the HPV vaccine in populations with low vaccine engagement. Small sample sizes did not allow for additional examination of the heterogeneity that may exist between the different counties or associated subgroups. Lastly, the latter portion of data collection took place during the COVID-19 pandemic, and it is unclear the extent to which this impacted parental and provider perceptions of vaccines and responses to this survey.

## 5. Conclusions

Our study offers considerations for developing community-level HPV vaccination campaigns among unvaccinated adolescents at parental, provider, and clinic levels and contributes to the limited body of data on the barriers and facilitators of HPV vaccination in at-risk rural communities of Western NC relying on public health clinics for vaccine services. This study provides a framework for larger studies with more participants in order to better characterize the thoughts and attitudes among rural North Carolinians. Health education of rural NC residents and providers in public health settings may identify future interventions to increase HPV vaccine uptake.

## Figures and Tables

**Table 1:** Characteristics of respondents.

Characteristic	Parent, N = 32 ^1^	Provider, N = 29 ^1^
Age	36 (31, 41)	37 (30, 50)
Unknown	3	0
Ethnicity		
Hispanic or Latino	5 (16%)	1 (3.6%)
Non-Hispanic or Latino	26 (81%)	27 (96%)
Other	1 (3.1%)	0 (0%)
Unknown	0	1
Race		
White alone	29 (91%)	27 (96%)
Black or African American	1 (3.1%)	0 (0%)
Multiracial	1 (3.1%)	0 (0%)
Other	1 (3.1%)	1 (3.6%)
Unknown	0	1
Gender		
Female	26 (84%)	27 (96%)
Male	5 (16%)	1 (3.6%)
Unknown	1	1
County		
Cherokee	5 (16%)	5 (17%)
Clay	6 (19%)	5 (17%)
Graham	5 (16%)	3 (10%)
Macon	7 (22%)	5 (17%)
McDowell	5 (16%)	5 (17%)
Polk	4 (12%)	5 (17%)
Yancey	0 (0%)	1 (3.4%)
Position, n (%)	Who Provided you with this survey today?	Number of Staff
MD/DO ^2^	0 (0)	2 (6.9)
Physician Assistant	0 (0)	1 (3.4)
Nurse Practitioner	0 (0)	3 (10)
Nurse	24 (75)	20 (69)
Front desk/scheduling	5 (16)	1 (3.4)
Other	3 (9.4)	2 (6.9)

^1^ Median (IQR); n (%); ^2^ MD, medical doctor; DO, doctor of osteopathic medicine.

**Table 2:** Provider and parent-reported characteristics in Western North Carolina health department clinics.

Provider/staff reported clinic characteristics	
What is the estimated patient volume per day in your practice setting?, Median (IQR)	35 (20–50)
Unknown	1
What is the estimated volume of patients that are seen for vaccines per day in your practice setting?, Median (IQR)	14 (10–20)
Unknown	1
Parent reported clinic characteristics	
How many times have you visited this clinic in the last year?	2 (1, 3)
Unknown	2
How many times have you visited this clinic for vaccinations?	2 (1, 2)
Unknown	2
Where do you get most of your health information?	
Friends or family	1 (3.1%)
Health Care Provider/Doctor’s Office	15 (47%)
Health Department	2 (6.2%)
Online/Internet	8 (25%)
Other	6 (19%)
The child attending the visit today is ___________.	
Female	20 (65%)
Male	11 (35%)
Unknown	1

**Table 3:** Parent-reported knowledge of HPV vaccination by sex of child.

Characteristic	Female, N = 20	Male, N = 11	*p*-Value ^1^
How effective do you think the HPV vaccine is in preventing genital warts? n (%)			0.18
Slightly effective	3 (15)	0 (0)	
Moderately	4 (20)	5 (62)	
Very	9 (45)	2 (25)	
Extremely effective	4 (20)	1 (12)	
Unknown	0	3	
How effective do you think the HPV vaccine is in preventing cancer of the genitals (penis, vulva, vagina, cervix), n (%)			0.79
Slightly effective	2 (10)	1 (11)	
Moderately	5 (25)	4 (44)	
Very	9 (45)	3 (33)	
Extremely effective	4 (20)	1 (11)	
Unknown	0	2	
How effective do you think the HPV vaccine is in preventing cancer of the anus?, n (%)			0.74
Slightly effective	2 (11)	2 (25)	
Moderately	5 (26)	3 (38)	
Very	8 (42)	2 (25)	
Extremely effective	4 (21)	1 (12)	
Unknown	1	3	

^1^ Fisher’s exact test; abbreviations: HPV, human papillomavirus.

## Data Availability

The dataset used in this study may be available upon reasonable request.
